# Brain Imaging of Nicotinic Receptors in Alzheimer's Disease

**DOI:** 10.4061/2010/548913

**Published:** 2010-12-28

**Authors:** Jin Wu, Masatomo Ishikawa, Jichun Zhang, Kenji Hashimoto

**Affiliations:** Division of Clinical Neuroscience, Center for Forensic Mental Health, Chiba University, 1-8-1 Inohana Chiba 260-8670, Japan

## Abstract

Neuronal nicotinic acetylcholine receptors (nAChRs) are a family of ligand-gated ion channels which are widely distributed in the human brain. Several lines of evidence suggest that two major subtypes (*α*4*β*2 and *α*7) of nAChRs play an important role in the pathophysiology of Alzheimer's disease (AD). Postmortem studies demonstrated alterations in the density of these subtypes of nAChRs in the brain of patients with AD. Currently, nAChRs are one of the most attractive therapeutic targets for AD. Therefore, several researchers have made an effort to develop novel radioligands that can be used to study quantitatively the distribution of these two subtypes in the human brain with positron emission tomography (PET) and single-photon emission computed tomography (SPECT). In this paper, we discuss the current topics on *in vivo* imaging of two subtypes of nAChRs in the brain of patients with AD.

## 1. Introduction

Alzheimer's disease (AD) is the most common neurodegenerative disorder in the elderly and has become a major worldwide health problem. Several reports indicated that it is affecting almost 1 in 10 individuals over the age of 65 [1], and as life expectancy increases, over 37 million people suffer with AD, and it is projected to quadruple by 2050 [2]. AD accounts for over 50% of senile dementia and the majority of presenile dementia cases and is characterized by progressive deterioration of higher cognitive functions including the loss of memory [3, 4].

van Duijn and Hofman [5] reported the inverse relationship between smoking history and early onset AD, suggesting that smoking may protect against AD [6]. Furthermore, Rusted and Trawley [7] reported acute improvements in prospective memory following nicotine administration. Although Swan and Lessov-Schlaggar [8] discuss the effects of tobacco smoke and nicotine on cognition in their review, smoking is associated with increased risk for negative preclinical and cognitive outcomes in younger people as well as in older adults. More recently, a meta-analysis including longitudinal studies published between 1995 and 2007 reported that current smokers relative to never-smokers were at increased risk of AD, vascular dementia, any dementia, and cognitive decline, in over the age of 65 [9]. Several lines of evidence demonstrated that smoking almost doubled the risk of AD and that smoking cessation might contribute to a reduction of risk factors for AD and cardiovascular disease [10, 11]. Noteworthy, the later is also known as a risk factor for AD. These results suggest that smoking cessation may play an important role in not only primary but also secondary prevention of AD. In contrast, although the discussion about neuroprotection by smoking has been continued, it is possible that nicotinic acetylcholine receptors (nAChRs) in the brain might play a role in the pathophysiology of AD. 

**Figure 1:**
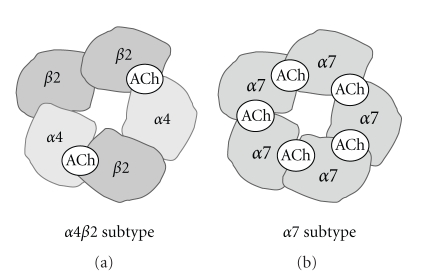
Structures of *α*4*β*2 nAChR (a) and *α*7 nAChR (b).

The nAChRs are one of the main classes of AChRs, which have a pentameric structure composed of five membrane spanning subunits, of which nine different types have thus far been identified and cloned. To date, twelve neuronal nAChR subunits have been described [12]; nine (*α*2–*α*10) code for subunits [12] based on the presence of adjacent cysteine residues in the predicted protein sequences, in a region homologous to the putative agonist-binding site of the muscle, a subunit (*α*1) and three referred to as non-*α* or *β*-subunits (*β*2–*β*4). Among the several nAChR subtypes in the human central nervous system (CNS), the heteromeric *α*4*β*2 and homomeric *α*7 subtypes (Figure 1) are predominant in the brain [13, 14]. It has been reported that other subtypes (e.g., *α*3, *α*6) exist in the brain [15, 16] and that *α*6 subtype might be mainly involved in the pathophysiology of Parkinson's disease [16]. Furthermore, studies using postmortem human brain samples have demonstrated alterations in the levels of *α*4 and *α*7 nAChR in the brains of patients with AD [15, 17–19]. Despite its lower number, loss of *α*3 subtype consistent with *α*4 and *α*7 nAChR subtypes was also observed in the brains of patients with AD [15]. Taken together, it is likely that these two subtypes (*α*4*β*2 and *α*7) of nAChR might play a role in the pathogenesis of AD. Therefore, it is of great interest to examine whether these two subtypes of nAChR are altered in the living brain of patients with AD using brain imaging techniques. 

In this paper, we discuss the recent findings on imaging of these two nAChRs (*α*4*β*2 and *α*7) in the brain with AD using positron emission tomography (PET) and single-photon emission computed tomography (SPECT).

## 2. *α*4*β*2 nAChRs Subtype

### 2.1. Relationship between Amyloid-*β* and *α*4*β*2 nAChR

 Amyloid *β* protein (A*β*) is a major constituent of senile plaques and one of the candidates for the cause of the neurodegeneration found in AD. It has been shown that the accumulation of A*β* precedes other pathological changes and causes neurodegeneration or neuronal death *in vitro and in vivo* [20, 21]. The loss of memory seen in AD is thought to be associated with A*β*-induced impairment of synaptic plasticity such as long-term potentiation (LTP) in the hippocampus. There are lines of evidence suggesting that nAChR activation provides protection against A*β*-induced neurotoxicity in cultured cortical neurons [22, 23]. These results indicated that nicotine protects against A*β*-induced neuronal death, and similar effect has been also observed in those selective *α*4*β*2 nAChR agonists such as cytosine and epibatidine, but this neuroprotection is blocked by the selective *α*4*β*2 nAChR antagonist dihydro-*β*-erythroidine (DH*β*E). Moreover, recently, Wu et al. [24] investigated a possible role of *α*4*β*2 nAChR in mediating the impairment of long-term potentiation (LTP) by various forms of A*β* in *in vivo*. They reported that intracerebroventricular injection of A*β*_40_, A*β*_25–35_, or A*β*_31–35_ significantly suppressed high-frequency stimulation-induced LTP. Similarly, epibatidine dose dependently suppressed the induction of LTP. Whereas DH*β*E showed no effect on the induction of LTP, it significantly reversed A*β*_31–35_-induced LTP impairment. These findings suggest that *α*4*β*2 nAChR, which can be directly activated by A*β*, is required for A*β* suppression of LTP *in vivo*. The mechanisms by which nicotine enhanced the inhibition of LTP by A*β* were not clear. A possible explanation is that nicotine could activate nAChRs present in inhibitory interneurons, thereby potentiating inhibitory inputs to hippocampal neurons.

### 2.2. Cognition and *α*4*β*2 nAChR Agonists

It is likely that reduced density of nAChR is related to dementia severity, assessed using a global rating. Nicotine has been postulated to be a possible treatment for AD, improving cognition in humans [25]. Recently, Loughead et al. [26] reported novel evidence that the *α*4*β*2 partial agonist varenicline increased working memory-related brain activity after 3 days of nicotine abstinence, particularly at high levels of task difficulty, with associated improvements in cognitive performance among highly dependent smokers.

### 2.3. Postmortem Studies of *α*4*β*2 nAChR in the Brain of Patients with AD

Not only transmitter release but also receptor-binding sites may be altered in the brain of AD patients [27–29]. Postmortem studies showed the reduction (up to 50%) of *α*4*β*2 subtype of nAChRs in brain of patients with AD [30], and it may occur very early in the course of AD [31]. Both *α*4 and *α*7 subunits are known to be important constituents in *α*4*β*2 and *α*7 receptor subtypes, respectively. Investigation using the autopsy samples of human cerebral cortex has clearly shown that these two subtypes (*α*4 and *α*7 isoforms) are significantly decreased in their protein amount in the cortices of AD patients [15, 19, 32].

### 2.4. Imaging of *α*4*β*2 nAChR Subtype

**Figure 2:**
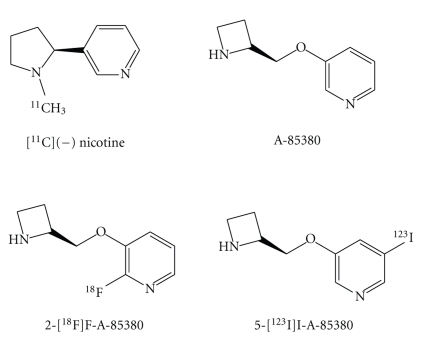
Chemical structures of radioligands for nAChRs.

Considering the role of *α*4*β*2 nAChR in the pathophysiology of AD, it is of great interest to study *α*4*β*2 nAChR in the living human brain using PET/SPECT. Much effort has been devoted to visualize *α*4*β*2 nAChR in the brain by PET/SPECT. Currently, two PET ligands, including [^11^C]nicotine and 2-[^18^F]fluoro-3-(2 (*S*)azetidinylmethoxy)pyridine (2-[^18^F]F-A-85380), and a SPECT ligand, 5-[^123^I]iodo-3-(2 (*S*)-2-azetidinylmethoxy)pyridine (5-[^123^I]I-A-85380), (Figure 2) for *in vivo* imaging of *α*4*β*2 nAChR in the human brain have been used in clinical studies [33–35].

### 2.5. [^11^C]Nicotine

The development of radiolabelled nicotine [36, 37] has allowed for evaluating the uptake and distribution of nAChR in the living human brain [38–40]. The data obtained by [^11^C]nicotine is generally consistent with the known pattern of nAChR measured by *in vitro* binding in autopsy brain tissue [39]. [^11^C]nicotine-PET has been used to study *α*4*β*2 nAChR in human brain, and a severe loss of the nAChR has been detected in the brain of patients with AD [13]. Cortical nAChRs in mild AD patients are robustly associated with the cognitive function of attention [35] and have revealed a significant negative correlation between severity of cognitive impairment and density of brain nAChR [40]. It will be, therefore, of interest to study an alteration in *α*4*β*2 nAChR at a presymptomatic stage of AD. Furthermore, the *in vivo* cortical AChE inhibition and [^11^C]nicotine binding were associated with changes in the attention domain of cognition rather than episodic memory when administering galantamine [41]. Thus, [^11^C]nicotine-PET may be also used for monitoring treatment efficacy in AD patients [41, 42].

Unfortunately, [^11^C]nicotine displays high levels of nonspecific binding, rapid metabolism, and rapid washout of the brain [43]. The heterogeneity of [^11^C]nicotine binding in the brain also precludes the identification of a reference region which may be used to accurately determine nonspecific binding. Taken together, it is unlikely that [^11^C]nicotine might be a suitable PET ligand for *in vivo* imaging of *α*4*β*2 nAChR in human brain.

### 2.6. 2-[^18^F]F-A-85380 and 5-[^123^I]I-A-85380

A-85380 [3-(2(*S*)-azetidinylmethoxy) pyridine] is a potent and selective agonist with high affinity for *α*4*β*2 nAChR subtype and low affinity for other nAChR subtypes [44]. A-85380 is effective in a wide range of preclinical models of CNS disorders [45, 46]. Recently, A-85380 was successfully labeled using ^18^F or ^125/123^I with a high affinity (*K*_*i*_ = 50 pM for F and *K*_*i*_ = 15 pM for I) for *α*4*β*2 nAChR [44, 47, 48]. These radioligands have been evaluated *in vitro* and *in vivo* as PET/SPECT radioligands to visualize *α*4*β*2 nAChR subtype in the brain [49, 50]. In healthy nonsmoking human brain, both 2-[^18^F]F-A85380 and 5-[^123^I]I-A85380 have revealed a pattern of highest uptake in the thalamus, intermediate in the midbrain, pons, cerebellum, and cortex, and lowest in white matter [50–52], which is consistent with the regional distribution of *α*4*β*2 nAChR. 

Furthermore, a study of age-related decline in nicotinic receptor availability showed that regional *β*2 nAChR availability were inversely correlated with decline ranging from 32% (thalamus) to 18% (occipital cortex) over the adult lifespan, or up to 5% per decade [53]. These results may corroborate postmortem reports of decline in high-affinity nicotine binding with age and may aid in elucidating the role of *β*2-nAChR in cognitive aging. In addition, 2-[^18^F]F-A-85380 or 5-[^123^I]I-A-85380 have been used to evaluate the effect of smoking on occupancy of *α*4*β*2 nAChR [54, 55]. Smoking 0.13 (1 to 2 puffs) of a cigarette resulted in 50% occupancy of *α*4*β*2 nAChR for 3.1 hours after smoking. Smoking a full cigarette (or more) resulted in more than 88% receptor occupancy and was accompanied by a reduction in cigarette craving. The extent of receptor occupancy found herein suggests that smoking may lead to withdrawal alleviation by maintaining nAChR in the desensitized state. 

**Figure 3:**
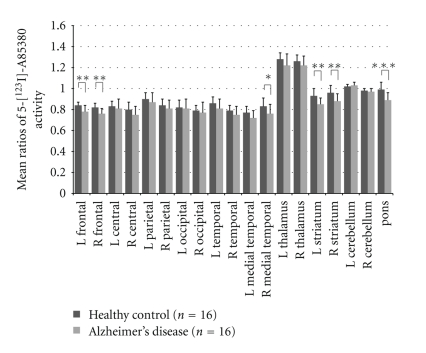
Comparison with regional uptake values of 5-[^123^I]I-A85380 in age-matched healthy control and patients with Alzheimer's disease. Significant bilateral reductions in nicotinic receptor binding were identified in frontal, striatal, right medial temporal, and pons in patients with AD compared to controls. (Data is from the paper of O'Brien et al. [59].) *P < .05, **P < .005, ***P < .001.

**Figure 4:**
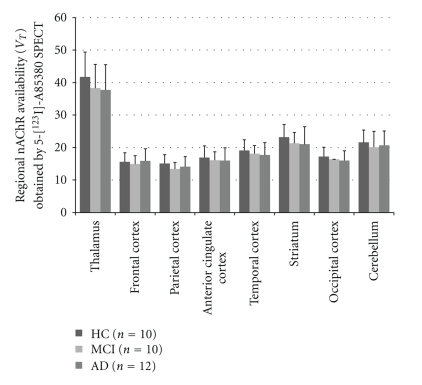
Regional *α*4*β*2-nAChR availability (*V*_*T*_) of 5-[^123^I]I-A85380 in age-matched healthy control (HC), mild cognitive impairment (MCI), and Alzheimer's disease (AD) groups. No significant regional differences among the subject groups for any of the 8 regions, including the 4 neocortical regions, were identified. (Data is from the paper of Mitsis et al. [58].)

Both 2-[^18^F]F-A-85380 and 5-[^123^I]I-A-85380 have been used in AD patients [51, 56–60]. In 17 patients with moderate to severe AD and 6 subjects with amnestic mild cognitive impairment (MCI) compared with 10 healthy control subjects, Sabri et al. [56] found significant reductions of *α*4*β*2 nAChR in brain regions (hippocampus, caudate, frontal cortex, temporal cortex, posterior cingulate, anterior cingulate, and parietal cortex) in the brain of AD by using 2-[^18^F]F-A-85380. Most recently, Kendziorra et al. [57] reported that both patients with AD and those with MCI showed a significant reduction in 2-[^18^F]F-A-85380 binding potential in typical AD-affected brain regions and that the 2-[^18^F]F-A-85380 binding potential correlated with the severity of cognitive impairment. In addition, only MCI patients who converted to AD in the later course (*n* = 5) had a reduction in 2-[^18^F]F-A-85380 binding potential. Thus, it is likely that 2-[^18^F]F-A-85380 PET might give prognostic information about a conversion from MCI to AD. Similar findings were also reported by 5-[^123^I]I-A-85380, showing significant reductions in the activity ratios of the region of interest to cerebellum in the frontal, striatal, right medial temporal, and pontine regions in 16 patients with AD compared with 16 healthy control subjects [59] (Figure 3). These findings suggest that a reduction in *α*4*β*2 nAChR occurs during symptomatic stages of AD and that the *α*4*β*2 nAChR availability in these regions correlated with the severity of cognitive impairment. In contrast, there were no differences in distribution volume (DV) of nAChR between the healthy controls and early AD patients (Figure 4) [51, 58]. 

**Figure 5:**
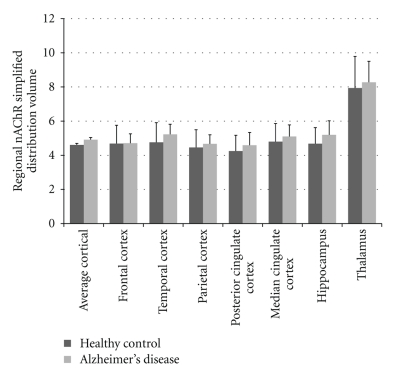
Regional nAChR simplified distribution volume (DV(s)) of 2-[^18^F]F-A85380 in healthy control (HC) and Alzheimer's disease (AD) groups. No significant difference in nAChR DV(s) was found between both groups. (Data is from the paper of Ellis et al. [51].)

2-[^18^F]F-A-85380 PET has been used to observe outcome of drug treatment for the improvements of cognition in patients with mild AD [61]. However, no significant correlations were found between cognitive measures and nAChR simplified DV (Figure 5). These results are similar to the results reported by Kadir et al. [41] in their studies using [^11^C]nicotine. The relationship between cognition in AD and cholinergic dysfunction may be related to a number of factors, including the degree of cholinergic system (or receptor) loss, the other nAChR subtypes, or other neurochemical systems.

## 3. *α*7 nAChR Subtype

### 3.1. Relationship between A*β* and *α*7 nAChR

**Figure 6:**
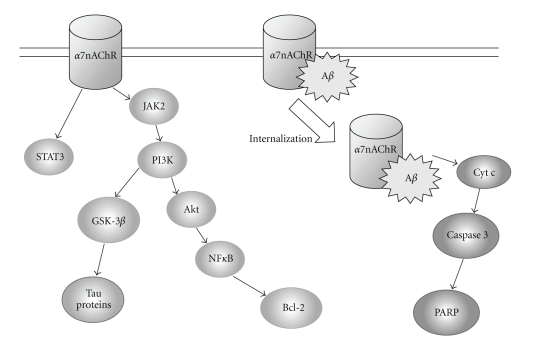
Schematic representation of neuroprotective role of *α*7 nAChR.

Of the two major subtypes of nAChRs in the CNS, *α*7 subtype has lower affinity for ACh compared to *α*4*β*2 subtype [62]. Accumulating evidence suggests that *α*7 nAChR plays a role in the pathophysiology of AD. A*β* has picomolar affinity for *α*7 nAChR [63, 64], which results in the formation of A*β*-*α*7 nAChR complex. This complex is known to move intracellularly and cause neurotoxicity [63–65]. Interestingly, this neurotoxicity is not present in transgenic mouse model of AD overexpressing a mutated form of the human amyloid precursor protein (APP) and lacking the *α*7 nAChR [66]. Recently, Bencherif and Lippiello [67] pointed out that the *α*7-JAK2-(NF-*κ*B; STAT3)-Bcl2 prosurvival pathway is important for the neuroprotective role of *α*7 nAChR (Figure 6). By blocking cytosolic cytochrome C, which is released from the mitochondria via A*β*_1–42_, Bcl2 fully counteracts the A*β*_1–42_-induced apoptosis of cells [68]. The fact that this antiapoptotic pathway is further related with ApoE4 [69], GSK-3*β*-activated tau phosphorylation [70], and Wnt signaling pathways [71] denotes the critical role of *α*7 nAChR in pathophysiology of AD.

The 3xTg-AD mice [72], which are triple transgenic mice expressing APP, presinilin-1, and Tau, were shown to have an age-dependent reduction of *α*7 nAChR. This reduction was limited to brain regions where intraneuronal A*β*_42_ accumulation occurred [73]. The early cognitive deficits of 3xTG-AD mice also correlate with intracellular A*β* accumulation, and the clearing of this A*β* accumulation by immunotherapy reverses the early cognitive impairment [74]. 

Tg2576 transgenic mice (APPswe) dramatically reduced A*β* plaque expression with chronic administration of nicotine for 5.5 months [75]. It is further reported that a 10-day administration of nicotine reduced the guanidinium-soluble A*β* levels by 46 to 66%, whereas the intracellular A*β* levels remained unchanged [76]. This treatment with nicotine also resulted in less glial fibrillary acidic protein- (GFAP-) immunoreactive astrocytes around the amyloid plaques and increased numbers of *α*7 nAChR in the cortex of APPswe mice [76]. Bencherif [68] points out the importance of these data, as reduction of A*β* with anti-A*β* antibody treatment is reported to rapidly recover the associated neuritic dystrophy in living animals [77].

Orr-Urtreger et al. [78] generated *α*7 nAChR gene knock-out (KO) mice, and the resulting *α*7 nAChR KO mice did not show any morphological central nervous system abnormalities [78, 79], but behavioral tests point out some cognitive deficits in KO mice, such as impaired sustained attention [80, 81], impairment in working memory [82], and impairment in performance under high attentional demand [83]. The cognitive deficits seen in APP transgenic mice worsen when *α*7 nAChR is absent at the same time [84]. These *α*7 nAChR KO APP mice showed significant reduction in hippocampal and basal forebrain choline acetyltransferase activity and loss of hippocampal neurons and markers; stereological analyses indicated more pronounced loss of hippocampal pyramidal neurons and volume loss compared with APP mice [84]. Taken all together, it is likely that *α*7 nAChR might play an important role in the process of A*β* disposition which was detected in the brain of patients with AD.

### 3.2. Cognition and *α*7 nAChR Agonists

A number of *α*7 nAChR agonists are reported to improve recognition memory in rodents. These agonists include tropisetron [85], ABBF [86], AR-R 17779 [87], SSR180711 [88, 89], A-582941 [90], and SEN123333 [91]. In nonhuman primates, improvements in long-delay performance of delayed matching tasks are reported by *α*7 nAChR agonists GTS-21 [92] and A-582941 [93]. 

It is reported that nicotine inhibits A*β* deposition and aggregation in the cortex and hippocampus of APP transgenic mice [94]. RNA interference experiments indicated that these nicotine-mediated effects require *α*7 nAChR. In another study [70], the selective *α*7 nAChR agonist A-582941 led to increased phosphorylation of the inhibitory regulating amino acid residue Ser-9 on glycogen synthase kinase 3*β* (GSK3*β*), a major kinase responsible for tau hyperphosphorylation in AD neuropathology. This was observed in mouse cingulate cortex and hippocampus and was not observed in *α*7 nAChR KO mice. S9-GSK3*β* phosphorylation was also seen in the hippocampus of Tg2576 (APP), as well as wild-type mice by steady-state exposure of A-582941. Moreover, continuous infusions of A-582941 decreased phosphorylation of tau in hippocampal CA3 Mossy fibers in a hypothermia-induced tau hyperphosphorylation mouse model and also decreased spinal motoneurons in AD double transgenic APP/tau mouse line. This group points out that *α*7 nAChR agonists may have therapeutic potential through GSK3*β* inhibition followed by reduction of tau hyperphosphorylation and further suggest that this pharmacology may have the potential to provide disease modifying benefit in the treatment of AD.

It is reported that the *α*7 nAChR agonist GTS-21 prevented A*β*_25–35_-induced impairment of acquisition performance and probe trail test in Morris water maze [95]. Their study showed first *in vivo *evidence that treatment with GTS-21 ameliorates the A*β*-induced deficit in spatial cognition through not only activating *α*7 nAChR but also preventing the A*β*-impaired *α*7 nAChR.

 Using a novel selective *α*7 nAChR partial agonist S 24795, Wang et al. [96] showed that, in contrast to anti-AD drugs, galantamine (a cholinesterase inhibitor) and memantine (an *N*-methyl-D-aspartate (NMDA) receptor antagonist), S 24795 reduced or limited A*β*_42_-*α*7 nAChR association, A*β*_42_-induced tau phosphorylation, A*β*_42_ accumulations, and A*β*_42_-mediated inhibition of *α*7 nAChR Ca^2+^ influx in rodent brain [96]. S 24795 more importantly restored *α*7 nAChR functional deficits which had resulted from continued exposure to exogenous A*β*_42_.

Taken all together, *α*7 nAChR is one of the therapeutic targets for AD [97, 98].

### 3.3. Postmortem Studies of *α*7 nAChR in the Brain of Patients with AD

In the postmortem brain of patients with AD, decline of *α*7 nAChR appears early in the disease and was associated with the progression of cognitive deficits [99–101]. Although the protein levels are reduced in the cortex and hippocampus of AD patients [15, 19, 32, 100, 102], contradictions arise at the level of gene transcription. For example, levels of *α*7 nAChR protein were reduced by 36% in the hippocampus of AD patients [15], but *α*7 nAChR mRNA expression is increased by 65% [18]. Furthermore, no differences in [^125^I]*α*-bungarotoxin binding were found in the frontal cortex of AD patients [103] and negative reduction of the *α*7 nAChR protein levels [104].

### 3.4. Imaging of *α*7 nAChR in the Brain

Given the role of *α*7 nAChR in the pathogenesis of AD, it is of great interest to study *α*7 nAChR in the living human brain using PET/SPECT. Much effort has been devoted to visualize *α*7 nAChR in the brain by PET/SPECT, but the development of a radioligand that depicts *α*7 nAChR specifically has been problematic due to its relatively low amount in the brain [105–108]. Generally, *α*-bungarotoxin and MLA are well known as specific *α*7 nAChR antagonists. However, due to their large molecular weights, they have difficulty passing through the blood-brain barrier which makes them unfavorable for radioligands [109–111]. Consequently, a number of radioligands for *α*7 nAChR are being developed and evaluated as PET/SPECT radioligand. However, all radioligands except [^11^C]CHIBA-1001 were unsuccessful [112].

### 3.5. [^11^C]CHIBA-1001 as a Novel PET Ligand for *α*7 nAChR

**Figure 7:**
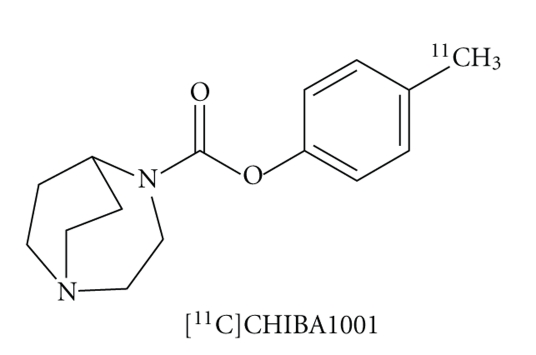
Chemical structure of [^11^C]CHIBA-1001.

We developed a novel PET ligand, 4-[^11^C]methylphenyl 1,4-diazabicyclo[3.2.2.]nonane-4-carboxylate ([^11^C]CHIBA-1001) (Figure 7). A PET study using conscious monkeys demonstrated that the distribution of radioactivity in the brain regions after intravenous administration of [^11^C]CHIBA-1001 was blocked by pretreatment with the selective *α*7 nAChR agonist SSR180711 (5.0 mg/kg), but not the selective *α*4*β*2 nAChR agonist A85380 (1.0 mg/kg) [89]. In addition, we reported that the order of drugs for the inhibition of [^3^H]CHIBA-1001 binding to rat brain membranes was similar to *α*7 nAChR pharmacological profiles [113]. We also reported a preliminary PET study of [^11^C]CHIBA-1001 in a healthy human [114, 115]. Very recently, we reported that [^125^I]CHIBA-1006, an iodine derivative of SSR180711, has a high affinity for *α*7 nAChR as compared with CHIBA-1001 [116]. Considering the good brain permeability of derivatives (e.g., SSR180711 and CHIBA-1001) of CHIBA-1006, it would be of great interest to examine whether [^123^I]CHIBA-1006 and [^124^I]CHIBA-1006 are suitable radioligands for *in vivo *labeling of *α*7 nAChRs in the brain using SPECT and PET, respectively [116].

At present, [^11^C]CHIBA-1001 is the only PET ligand which can be available for *in vivo* study of *α*7 nAChRs in intact human brain [114]. PET studies of [^11^C]CHIBA-1001 in patients with AD are currently underway with [^11^C]Pittsburgh compound D ([^11^C]PiB)-PET and [^18^F]fluorodeoxyglucose ([^18^F]FDG)-PET. This study aims to evaluate the relationship between the distribution of *α*7 nAChR (assessed by [^11^C]CHIBA-1001) and A*β* disposition (assessed by [^11^C]PiB), while estimating the stage and cognitive levels (assessed by [^18^F]FDG-PET and neuropsychological examinations) for each AD patient.

## 4. Conclusions

Considering the importance of early prevention of onset of AD, it is very important to detect alternations in nAChRs at the presymptomatic stage of AD. In patients with MCI, the early detection and early therapeutic intervention would be beneficial. Therefore, brain imaging of nAChRs using PET and SPECT will be a powerful tool to study the mechanisms underlying pathological brain processes of cognitive disturbances in these patients. Currently, some PET and SPECT ligands for both subtypes (*α*4*β*2 nAChR and *α*7 nAChR) have been used to investigate the changes in receptor densities and functions of patients with AD. Gaining a better understanding of the role of nAChRs in the pathophysiology of AD is expected to provide new perspectives for treating this disorder.

##  Conflict of Interests 

The authors have no conflict of interests.

## Abbreviations

AD: Alzheimer's disease
DV: Distribution volume
GFAP: Glial fibrillary acidic protein
LTP: Long-term potentiation
nAChR: Nicotinic acetylcholine receptor
PET: Positron emission tomography
SPECT: Single-photon emission tomography.

## References

[1] Evans DA, Funkenstein HH, Albert MS (1989). Prevalence of Alzheimer’s disease in a community population of older persons. Higher than previously reported. *Journal of the American Medical Association*.

[2] Brookmeyer R, Johnson E, Ziegler-Graham K, Arrighi HM (2007). Forecasting the global burden of Alzheimer’s disease. *Alzheimer’s and Dementia*.

[3] Octave JN (1995). The amyloid peptide and its precursor in Alzheimer’s disease. *Reviews in the Neurosciences*.

[4] Grober E, Hall CB, Lipton RB, Zonderman AB, Resnick SM, Kawas C (2008). Memory impairment, executive dysfunction, and intellectual decline in preclinical Alzheimer’s disease. *Journal of the International Neuropsychological Society*.

[5] van Duijn CM, Hofman A (1991). Relation between nicotine intake and Alzheimer’s disease. *British Medical Journal*.

[6] Graves AB, van Duijn CM, Chandra V (1991). Alcohol and tobacco consumption as risk factors for Alzheimer's disease: a collaborative re-analysis of case-control studies. *International Journal of Epidemiology*.

[7] Rusted JM, Trawley S (2006). Comparable effects of nicotine in smokers and nonsmokers on a prospective memory task. *Neuropsychopharmacology*.

[8] Swan GE, Lessov-Schlaggar CN (2007). The effects of tobacco smoke and nicotine on cognition and the brain. *Neuropsychology Review*.

[9] Peters R, Poulter R, Warner J, Beckett N, Burch L, Bulpitt C (2008). Smoking, dementia and cognitive decline in the elderly, a systematic review. *BMC Geriatrics*.

[10] Cataldo JK, Prochaska JJ, Glantz SA (2010). Cigarette smoking is a risk factor for Alzheimer’s disease: an analysis controlling for tobacco industry affiliation. *Journal of Alzheimer’s Disease*.

[11] Cataldo JK, Glantz SA (2010). Smoking cessation and Alzheimer’s disease: facts, fallacies and promise. *Expert Review of Neurotherapeutics*.

[12] Gotti C, Zoli M, Clementi F (2006). Brain nicotinic acetylcholine receptors: native subtypes and their relevance. *Trends in Pharmacological Sciences*.

[13] Paterson D, Nordberg A (2000). Neuronal nicotinic receptors in the human brain. *Progress in Neurobiology*.

[14] Dani JA, Bertrand D (2007). Nicotinic acetylcholine receptors and nicotinic cholinergic mechanisms of the central nervous system. *Annual Review of Pharmacology and Toxicology*.

[15] Guan ZZ, Zhang X, Ravid R, Nordberg A (2000). Decreased protein levels of nicotinic receptor subunits in the hippocampus and temporal cortex of patients with Alzheimer’s disease. *Journal of Neurochemistry*.

[16] Perez XA, Bordia T, McIntosh JM, Quik M (2010). *α*6*β*2* and *α*4*β*2* nicotinic receptors both regulate dopamine signaling with increased nigrostriatal damage: relevance to Parkinson's disease. *Molecular Pharmacology*.

[17] Perry EK, Morris CM, Court JA (1995). Alteration in nicotine binding sites in Parkinson's disease, Lewy body dementia and Alzheimer's disease: possible index of early neuropathology. *Neuroscience*.

[18] Hellström-Lindahl E, Mousavi M, Zhang X, Ravid R, Nordberg A (1999). Regional distribution of nicotinic receptor subunit mRNAs in human brain: comparison between Alzheimer and normal brain. *Molecular Brain Research*.

[19] Burghaus L, Schütz U, Krempel U (2000). Quantitative assessment of nicotinic acetylcholine receptor proteins in the cerebral cortex of Alzheimer patients. *Molecular Brain Research*.

[20] Yankner BA, Duffy LK, Kirschner DA (1990). Neurotrophic and neurotoxic effects of amyloid *β* protein: reversal by tachykinin neuropeptides. *Science*.

[21] Kowall NW, Beal MF, Busciglio J, Duffy LK, Yankner BA (1991). An *in vivo* model for the neurodegenerative effects of *β* amyloid and protection by substance P. *Proceedings of the National Academy of Sciences of the United States of America*.

[22] Kihara T, Shimohama S, Urushitani M (1998). Stimulation of *α*4*β*2 nicotinic acetylcholine receptors inhibits *β*- amyloid toxicity. *Brain Research*.

[23] Fu W, Jhamandas JH (2003). *β*-amyloid peptide activates non-*α*7 nicotinic acetylcholine receptors in rat basal forebrain neurons. *Journal of Neurophysiology*.

[24] Wu MN, He YX, Guo F, Qi JS (2008). *α*4*β*2 nicotinic acetylcholine receptors are required for the amyloid *β* protein-induced suppression of long-term potentiation in rat hippocampal CA1 region *in vivo*. *Brain Research Bulletin*.

[25] Newhouse PA, Potter A, Singh A (2004). Effects of nicotinic stimulation on cognitive performance. *Current Opinion in Pharmacology*.

[26] Loughead J, Ray R, Wileyto EP (2010). Effects of the *α*4*β*2 partial agonist varenicline on brain activity and working memory in abstinent smokers. *Biological Psychiatry*.

[27] Flynn DD, Mash DC (1986). Characterization of L-[^3^H]nicotine binding in human cerebral cortex: comparison between Alzheimer’s disease and the normal. *Journal of Neurochemistry*.

[28] Whitehouse PJ, Martino AM, Antuono PG (1986). Nicotinic acetylcholine binding sites in Alzheimer's disease. *Brain Research*.

[29] Whitehouse PJ, Martino AM, Wagster MV (1988). Reductions in [^3^H]nicotinic acetylcholine binding in Alzheimer’s disease and Parkinson’s disease: an autoradiographic study. *Neurology*.

[30] Warpman U, Nordberg A (1995). Epibatidine and ABT 418 reveal selective losses of *α*4*β*2 nicotinic receptors in Alzheimer brains. *NeuroReport*.

[31] Marutle A, Warpman U, Bogdanovic N, Lannfelt L, Nordberg A (1999). Neuronal nicotinic receptor deficits in Alzheimer patients with the Swedish amyloid precursor protein 670/671 mutation. *Journal of Neurochemistry*.

[32] Wevers A, Burghaus L, Moser N (2000). Expression of nicotinic acetylcholine receptors in Alzheimer’s disease: postmortem investigations and experimental approaches. *Behavioural Brain Research*.

[33] Gallezot JD, Bottlaender M, Grégoire MC (2005). *in vivo* imaging of human cerebral nicotinic acetylcholine receptors with 2-^18^F-fluoro-A-85380 and PET. *Journal of Nuclear Medicine*.

[34] Brody AL, Mandelkern MA, London ED (2006). Cigarette smoking saturates brain *α*4*β*2 nicotinic acetylcholine receptors. *Archives of General Psychiatry*.

[35] Kadir A, Almkvist O, Wall A, Långström B, Nordberg A (2006). PET imaging of cortical C-nicotine binding correlates with the cognitive function of attention in Alzheimer’s disease. *Psychopharmacology*.

[36] Maziere M, Comar D, Marazano C, Berger G (1976). Nicotine ^11^C: synthesis and distribution kinetics in animals. *European Journal of Nuclear Medicine*.

[37] Halldin C, Nagren K, Swahn CG, Langstrom B, Nyback H (1992). (*S*)- and (*R*)-[^11^C]nicotine and the metabolite (*R/S*)-[^11^C]cotinine. Preparation, metabolite studies and *in vivo* distribution in the human brain using PET. *International Journal of Radiation Applications and Instrumentation B*.

[38] Nyback H, Nordberg A, Langstrom B (1989). Attempts to visualize nicotinic receptors in the brain of monkey and man by positron emission tomography. *Progress in Brain Research*.

[39] Nordberg A, Nilsson-Hakansson L, Adem A (1989). The role of nicotinic receptors in the pathophysiology of Alzheimer’s disease. *Progress in Brain Research*.

[40] Nordberg A, Lundqvist H, Hartvig P, Lilja A, Langstrom B (1995). Kinetic analysis of regional (S)(-)^11^C-nicotine binding in normal and Alzheimer brains—*in vivo* assessment using positron emission tomography. *Alzheimer Disease and Associated Disorders*.

[41] Kadir A, Darreh-Shori T, Almkvist O (2008). PET imaging of the *in vivo* brain acetylcholinesterase activity and nicotine binding in galantamine-treated patients with AD. *Neurobiology of Aging*.

[42] Nordberg A, Lundqvist H, Hartvig P (1997). Imaging of nicotinic and muscarinic receptors in Alzheimer's disease: effect of tacrine treatment. *Dementia and Geriatric Cognitive Disorders*.

[43] Grunwald F, Biersack HJ, Kuschinsky W, Sorger D, Kampfer I, Knapp WH (1996). Nicotine receptor mapping. *European Journal of Nuclear Medicine*.

[44] Sullivan JP, Donnelly-Roberts D, Briggs CA (1996). A-85380 [3-(2(*S*)-azetidinylmethoxy) pyridine]: *in vitro* pharmacological properties of a novel, high affinity *α*4*β*2 nicotinic acetylcholine receptor ligand. *Neuropharmacology*.

[45] Rueter LE, Meyer MD, Decker MW (2000). Spinal mechanisms underlying A-85380-induced effects on acute thermal pain. *Brain Research*.

[46] Buckley MJ, Surowy C, Meyer M, Curzon P (2004). Mechanism of action of A-85380 in an animal model of depression. *Progress in Neuro-Psychopharmacology and Biological Psychiatry*.

[47] Rueter LE, Donnelly-Roberts DL, Curzon P, Briggs CA, Anderson DJ, Bitner RS (2006). A-85380: a pharmacological probe for the preclinical and clinical investigation of the *αβ* neuronal nicotinic acetylcholine receptor. *CNS Drug Reviews*.

[48] Mukhin AG, Gündisch D, Horti AG (2000). 5-iodo-a-85380, an *α*4*β*2 subtype-selective ligand for nicotinic acetylcholine receptors. *Molecular Pharmacology*.

[49] Chefer SI, London ED, Koren AO (2003). Graphical analysis of 2-[^18^F]FA binding to nicotinic acetylcholine receptors in rhesus monkey brain. *Synapse*.

[50] Kimes AS, Horti AG, London ED (2003). 2-[^18^F]F-A-85380: PET imaging of brain nicotinic acetylcholine receptors and whole body distribution in humans. *FASEB Journal*.

[51] Ellis JR, Villemagne VL, Nathan PJ (2008). Relationship between nicotinic receptors and cognitive function in early Alzheimer’s disease: a 2-[^18^F]fluoro-A-85380 PET study. *Neurobiology of Learning and Memory*.

[52] Fujita M, Ichise M, van Dyck CH (2003). Quantification of nicotinic acetylcholine receptors in human brain using [^123^I]5-I-A-85380 SPET. *European Journal of Nuclear Medicine and Molecular Imaging*.

[53] Mitsis EM, Cosgrove KP, Staley JK (2009). Age-related decline in nicotinic receptor availability with [^123^I]5-IA-85380 SPECT. *Neurobiology of Aging*.

[54] Cosgrove KP, Batis J, Bois F (2009). *β*2-nicotinic acetylcholine receptor availability during acute and prolonged abstinence from tobacco smoking. *Archives of General Psychiatry*.

[55] Brody AL, Mandelkern MA, Costello MR (2009). Brain nicotinic acetylcholine receptor occupancy: effect of smoking a denicotinized cigarette. *International Journal of Neuropsychopharmacology*.

[56] Sabri O, Kendziorra K, Wolf H, Gertz HJ, Brust P (2008). Acetylcholine receptors in dementia and mild cognitive impairment. *European Journal of Nuclear Medicine and Molecular Imaging*.

[57] Kendziorra K, Wolf H, Meyer PM Decreased cerebral *α*4*β*2* nicotinic acetylcholine receptor availability in patients with mild cognitive impairment and Alzheimer's disease assessed with positron emission tomography.

[58] Mitsis EM, Reech KM, Bois F (2009). ^123^I-5-IA-85380 SPECT imaging of nicotinic receptors in Alzheimer disease and mild cognitive impairment. *Journal of Nuclear Medicine*.

[59] O’Brien JT, Colloby SJ, Pakrasi S (2007). *α*4*β*2 nicotinic receptor status in Alzheimer’s disease using I-5IA-85380 single-photon-emission computed tomography. *Journal of Neurology, Neurosurgery and Psychiatry*.

[60] Colloby SJ, Perry EK, Pakrasi S (2010). Nicotinic ^123^I-5IA-85380 single photon emission computed tomography as a predictor of cognitive progression in alzheimer’s disease and dementia with lewy Bodies. *American Journal of Geriatric Psychiatry*.

[61] Ellis JR, Nathan PJ, Villemagne VL (2009). Galantamine-induced improvements in cognitive function are not related to alterations in *α*4*β*2 nicotinic receptors in early Alzheimer’s disease as measured *in vivo* by 2-[^18^F]Fluoro-A- 85380 PET. *Psychopharmacology*.

[62] Clarke PBS (1992). The fall and rise of neuronal *α*-bungarotoxin binding proteins. *Trends in Pharmacological Sciences*.

[63] Wang HY, Lee DHS, D’Andrea MR, Peterson PA, Shank RP, Reitz AB (2000). *β*-Amyloid(1–42) binds to *α*7 nicotinic acetylcholine receptor with high affinity. Implications for Alzheimer’s disease pathology. *Journal of Biological Chemistry*.

[64] Wang HY, Lee DHS, Davis CB, Shank RP (2000). Amyloid peptide A*β*(1–42) binds selectively and with picomolar affinity to *α*7 nicotinic acetylcholine receptors. *Journal of Neurochemistry*.

[65] D’Andrea MR, Nagele RG, Wang HY, Peterson PA, Lee DHS (2001). Evidence that neurones accumulating amyloid can undergo lysis to form amyloid plaques in Alzheimer’s disease. *Histopathology*.

[66] Dziewczapolski G, Glogowski CM, Masliah E, Heinemann SF (2009). Deletion of the *α*7 nicotinic acetylcholine receptor gene improves cognitive deficits and synaptic pathology in a mouse model of Alzheimer’s disease. *Journal of Neuroscience*.

[67] Bencherif M, Lippiello PM (2010). *α*7 neuronal nicotinic receptors: the missing link to understanding Alzheimer’s etiopathology?. *Medical Hypotheses*.

[68] Bencherif M (2009). Neuronal nicotinic receptors as novel targets for inflammation and neuroprotection: mechanistic considerations and clinical relevance. *Acta Pharmacologica Sinica*.

[69] Eddins D, Klein RC, Yakel JL, Levin ED (2009). Hippocampal infusions of apolipoprotein E peptides induce long-lasting cognitive impairment. *Brain Research Bulletin*.

[70] Bitner RS, Nikkel AL, Markosyan S, Otte S, Puttfarcken P, Gopalakrishnan M (2009). Selective *α*7 nicotinic acetylcholine receptor activation regulates glycogen synthase kinase3*β* and decreases tau phosphorylation *in vivo*. *Brain Research*.

[71] Farías GG, Vallés AS, Colombres M (2007). Wnt-7a induces presynaptic colocalization of *α*7-nicotinic acetylcholine receptors and adenomatous polyposis coli in hippocampal neurons. *Journal of Neuroscience*.

[72] Oddo S, Caccamo A, Shepherd JD (2003). Triple-transgenic model of Alzheimer’s disease with plaques and tangles: intracellular A*β* and synaptic dysfunction. *Neuron*.

[73] Oddo S, Caccamo A, Green KN (2005). Chronic nicotine administration exacerbates tau pathology in a transgenic model of Alzheimer’s disease. *Proceedings of the National Academy of Sciences of the United States of America*.

[74] Billings LM, Oddo S, Green KN, McGaugh JL, LaFerla FM (2005). Intraneuronal A*β* causes the onset of early Alzheimer’s disease-related cognitive deficits in transgenic mice. *Neuron*.

[75] Nordberg A, Hellström-Lindahl E, Lee M (2002). Chronic nicotine treatment reduces *β*-amyloidosis in the brain of a mouse model of Alzheimer’s disease (APPsw). *Journal of Neurochemistry*.

[76] Unger C, Svedberg MM, Yu WF, Hedberg MM, Nordberg A (2006). Effect of subchronic treatment of memantine, galantamine, and nicotine in the brain of Tg2576 (APPswe) transgenic mice. *Journal of Pharmacology and Experimental Therapeutics*.

[77] Brendza RP, Bacskai BJ, Cirrito JR (2005). Anti-A*β* antibody treatment promotes the rapid recovery of amyloid-associated neuritic dystrophy in PDAPP transgenic mice. *Journal of Clinical Investigation*.

[78] Orr-Urtreger A, Goldner FM, Saeki M (1997). Mice deficient in the *α*7 neuronal nicotinic acetylcholine receptor lack *α*-bungarotoxin binding sites and hippocampal fast nicotinic currents. *Journal of Neuroscience*.

[79] Paylor R, Nguyen M, Crawley JN, Patrick J, Beaudet A, Orr-Urtreger A (1998). *α*7 nicotinic receptor subunits are not necessary for hippocampal- dependent learning or sensorimotor gating: a behavioral characterization of Acra7-deficient mice. *Learning and Memory*.

[80] Hoyle E, Genn RF, Fernandes C, Stolerman IP (2006). Impaired performance of *α*7 nicotinic receptor knockout mice in the five-choice serial reaction time task. *Psychopharmacology*.

[81] Young JW, Crawford N, Kelly JS (2007). Impaired attention is central to the cognitive deficits observed in alpha 7 deficient mice. *European Neuropsychopharmacology*.

[82] Fernandes C, Hoyle E, Dempster E, Schalkwyk LC, Collier DA (2006). Performance deficit of *α*7 nicotinic receptor knockout mice in a delayed matching-to-place task suggests a mild impairment of working/episodic-like memory. *Genes, Brain and Behavior*.

[83] Keller JJ, Keller AB, Bowers BJ, Wehner JM (2005). Performance of *α*7 nicotinic receptor null mutants is impaired in appetitive learning measured in a signaled nose poke task. *Behavioural Brain Research*.

[84] Hernandez CM, Kayed R, Zheng H, Sweatt JD, Dineley KT (2010). Loss of *α*7 nicotinic receptors enhances *β*-amyloid oligomer accumulation, exacerbating early-stage cognitive decline and septohippocampal pathology in a mouse model of Alzheimer’s disease. *Journal of Neuroscience*.

[85] Hashimoto K, Fujita Y, Ishima T, Hagiwara H, Iyo M (2006). Phencyclidine-induced cognitive deficits in mice are improved by subsequent subchronic administration of tropisetron: role of *α*7 nicotinic receptors. *European Journal of Pharmacology*.

[86] Boess FG, de Vry J, Erb C (2007). The novel *α*7 nicotinic acetylcholine receptor agonist N-[(3*R*)-1-azabicyclo[2.2.2]oct-3-yl]-7-[2-(methoxy)phenyl]-1-benzofuran-2- carboxamide improves working and recognition memory in rodents. *Journal of Pharmacology and Experimental Therapeutics*.

[87] van Kampen M, Selbach K, Schneider R, Schiegel E, Boess F, Schreiber R (2004). AR-R 17779 improves social recognition in rats by activation of nicotinic *α*7 receptors. *Psychopharmacology*.

[88] Pichat P, Bergis OE, Terranova JP (2007). SSR180711, a novel selective *α*7 nicotinic receptor partial agonist: (II) efficacy in experimental models predictive of activity against cognitive symptoms of schizophrenia. *Neuropsychopharmacology*.

[89] Hashimoto K, Nishiyama S, Ohba H (2008). [^11^C]CHIBA-1001 as a novel PET ligand for *α*7 nicotinic receptors in the brain: a PET study in conscious monkeys. *PLoS ONE*.

[90] Tietje KR, Anderson DJ, Bitner RS (2008). Preclinical characterization of A-582941: a novel *α*7 neuronal nicotinic receptor agonist with broad spectrum cognition-enhancing properties. *CNS Neuroscience and Therapeutics*.

[91] Roncarati R, Scali C, Comery TA (2009). Procognitive and neuroprotective activity of a novel *α*7 nicotinic acetylcholine receptor agonist for treatment of neurodegenerative and cognitive disorders. *Journal of Pharmacology and Experimental Therapeutics*.

[92] Briggs CA, Anderson DJ, Brioni JD (1997). Functional characterization of the novel neuronal nicotinic acetylcholine receptor ligand GTS-21 *in vitro* and *in vivo*. *Pharmacology Biochemistry and Behavior*.

[93] Buccafusco JJ, Terry AV, Decker MW, Gopalakrishnan M (2007). Profile of nicotinic acetylcholine receptor agonists ABT-594 and A-582941, with differential subtype selectivity, on delayed matching accuracy by young monkeys. *Biochemical Pharmacology*.

[94] Liu Q, Zhang J, Zhu H, Qin C, Chen Q, Zhao B (2007). Dissecting the signaling pathway of nicotine-mediated neuroprotection in a mouse Alzheimer disease model. *FASEB Journal*.

[95] Chen L, Wang H, Zhang Z (2010). DMXB (GTS-21) ameliorates the cognitive deficits in beta amyloid_25-35_-injected mice through preventing the dysfunction of *α*7 nicotinic receptor. *Journal of Neuroscience Research*.

[96] Wang HY, Bakshi K, Shen C, Frankfurt M, Trocmé-Thibierge C, Morain P (2010). S 24795 limits *β*-amyloid-*α*7 nicotinic receptor interaction and reduces Alzheimer’s disease-like pathologies. *Biological Psychiatry*.

[97] Hashimoto K, Iyo M (2002). Amyloid cascade hypothesis of Alzheimer's disease and *α*7 nicotinic receptor. *Nihon Shinkei Seishin Yakurigaku Zasshi*.

[98] Toyohara J, Hashimoto K (2010). *α*7 nicotinic receptor agonists: potential therapeutic drugs for treatment of cognitive impairments in schizophrenia and Alzheimer's disease. *Open Medicinal Chemistry Journal*.

[99] Nordberg A (1994). Human nicotinic receptors—their role in aging and dementia. *Neurochemistry International*.

[100] Nordberg A (2001). Nicotinic receptor abnormalities of Alzheimer’s disease: therapeutic implications. *Biological Psychiatry*.

[101] Whitehouse PJ, Kalaria RN (1995). Nicotinic receptors and neurodegenerative dementing diseases: basic research and clinical implications. *Alzheimer Disease and Associated Disorders*.

[102] Martin-Ruiz CM, Court JA, Molnar E (1999). *α*4 but not *α*3 and *α*7 nicotinic acetylcholine receptor subunits are lost from the temporal cortex in Alzheimer’s disease. *Journal of Neurochemistry*.

[103] Davies P, Feisullin S (1981). Postmortem stability of *α*-bungarotoxin binding sites in mouse and human brain. *Brain Research*.

[104] Engidawork E, Gulesserian T, Balic N, Cairns N, Lubec G (2001). Changes in nicotinic acetylcholine receptor subunits expression in brain of patients with Down syndrome and Alzheimer's disease. *Journal of Neural Transmission, Supplement*.

[105] Falk L, Nordberg A, Seiger A, Kjaeldgaard A, Hellström-Lindahl E (2003). Higher expression of *α*7 nicotinic acetylcholine receptors in human fetal compared to adult brain. *Developmental Brain Research*.

[106] Marutle A, Zhang X, Court J (2001). Laminar distribution of nicotinic receptor subtypes in cortical regions in schizophrenia. *Journal of Chemical Neuroanatomy*.

[107] Court JA, Perry EK, Spurden D (1995). The role of the cholinergic system in the development of the human cerebellum. *Developmental Brain Research*.

[108] Court J, Martin-Ruiz C, Piggott M, Spurden D, Griffiths M, Perry E (2001). Nicotinic receptor abnormalities in Alzheimer’s disease. *Biological Psychiatry*.

[109] James RW, Bersinger NA, Schwendimann B, Fulpius BW (1980). Characterization of iodinated derivatives of *α*-bungarotoxin. *Hoppe-Seyler’s Zeitschrift fur Physiologische Chemie*.

[110] Davies ARL, Hardick DJ, Blagbrough IS, Potter BVL, Wolstenholme AJ, Wonnacott S (1999). Characterisation of the binding of [^3^H]methyllycaconitine: a new radioligand for labelling *α*7-type neuronal nicotinic acetylcholine receptors. *Neuropharmacology*.

[111] Navarro HA, Zhong D, Abraham P, Xu H, Carroll FI (2000). Synthesis and pharmacological characterization of [^125^I]iodomethyllycaconitine ([^125^I]iodo-MLA). A new ligand for the *α*(7) nicotinic acetylcholine receptor. *Journal of Medicinal Chemistry*.

[112] Toyohara J, Wu J, Hashimoto K (2010). Recent development of radioligands for imaging *α*7 nicotinic acetylcholine receptors in the brain. *Current Topics in Medicinal Chemistry*.

[113] Tanibuchi Y, Wu J, Toyohara J, Fujita Y, Iyo M, Hashimoto K (2010). Characterization of [^3^H]CHIBA-1001 binding to *α*7 nicotinic acetylcholine receptors in the brain from rat, monkey, and human. *Brain Research*.

[114] Toyohara J, Sakata M, Wu J (2009). Preclinical and the first clinical studies on [^11^C]CHIBA-1001 for mapping *α*7 nicotinic receptors by positron emission tomography. *Annals of Nuclear Medicine*.

[115] Sakata M, Wu J, Toyohara J Biodistribution and radiation dosimetry of the *α*7 nicotinic acetylcholine receptor ligand [^11^C]CHIBA-1001 in humans.

[116] Wu J, Toyohara J, Tanibuchi Y (2010). Pharmacological characterization of [^125^I]CHIBA-1006 binding, a new radioligand for *α*7 nicotinic acetylcholine receptors, to rat brain membranes. *Brain Research*.

